# Supplementary material for: COZOID: contact zone identifier for visual analysis of protein-protein interactions
Source: BMC Bioinformatics. 2018 Apr 6;19:125. doi: 10.1186/s12859-018-2113-6 (PMC5889581; doi:10.1186/s12859-018-2113-6)
Supplement: Supplementary file 2 — Software build. Executable binary file of the software tool. (ZIP 118,784 kb) [file 12859_2018_2113_MOESM2_ESM.zip › cozoid/java/docs/beansbinding-1.2.1-doc.zip!/org/jdesktop/el/impl/ValueExpressionImpl.html]

ValueExpressionImpl (Beans Binding)


---


|  |  |  |
| --- | --- | --- |
| **Overview** \| **Package** \| **Class** \| **Use** \| **Tree** \| **Deprecated** \| **Index** \| **Help** | |  |
| **PREV CLASS**   **NEXT CLASS** | **FRAMES**    **NO FRAMES**     **All Classes** |  |
| SUMMARY: NESTED \| FIELD \| CONSTR \| METHOD | DETAIL: FIELD \| CONSTR \| METHOD |  |


---


## org.jdesktop.el.impl Class ValueExpressionImpl

```
java.lang.Object
  org.jdesktop.el.Expression
      org.jdesktop.el.ValueExpression
          org.jdesktop.el.impl.ValueExpressionImpl
```

**All Implemented Interfaces:**: java.io.Externalizable, java.io.Serializable

---

``` public final class ValueExpressionImpl extends ValueExpression implements java.io.Externalizable ```

An `Expression` that can get or set a value.

In previous incarnations of this API, expressions could only be read.
`ValueExpression` objects can now be used both to retrieve a
value and to set a value. Expressions that can have a value set on them are
referred to as l-value expressions. Those that cannot are referred to as
r-value expressions. Not all r-value expressions can be used as l-value
expressions (e.g. `"${1+1}"` or
`"${firstName} ${lastName}"`). See the EL Specification for
details. Expressions that cannot be used as l-values must always return
`true` from `isReadOnly()`.

`The ExpressionFactory.createValueExpression(org.jdesktop.el.ELContext, java.lang.String, java.lang.Class ) method
can be used to parse an expression string and return a concrete instance
of ValueExpression that encapsulates the parsed expression.
The FunctionMapper is used at parse time, not evaluation time,
so one is not needed to evaluate an expression using this class.
However, the ELContext is needed at evaluation time.`

The `getValue(org.jdesktop.el.ELContext)`, `setValue(org.jdesktop.el.ELContext, java.lang.Object)`, `isReadOnly(org.jdesktop.el.ELContext)` and
`getType(org.jdesktop.el.ELContext)` methods will evaluate the expression each time they are
called. The `ELResolver` in the `ELContext` is used to
resolve the top-level variables and to determine the behavior of the
`.` and `[]` operators. For any of the four methods,
the `ELResolver.getValue(org.jdesktop.el.ELContext, java.lang.Object, java.lang.Object)` method is used to resolve all properties
up to but excluding the last one. This provides the `base`
object. At the last resolution, the `ValueExpression` will
call the corresponding `ELResolver.getValue(org.jdesktop.el.ELContext, java.lang.Object, java.lang.Object)`,
`ELResolver.setValue(org.jdesktop.el.ELContext, java.lang.Object, java.lang.Object, java.lang.Object)`, `ELResolver.isReadOnly(org.jdesktop.el.ELContext, java.lang.Object, java.lang.Object)` or
`ELResolver.getType(org.jdesktop.el.ELContext, java.lang.Object, java.lang.Object)` method, depending on which was called on
the `ValueExpression`.

See the notes about comparison, serialization and immutability in
the `Expression` javadocs.

**See Also:**: `javax.el.ELResolver`, `javax.el.Expression`, `javax.el.ExpressionFactory`, `javax.el.ValueExpression`, Serialized Form

---

**Nested Class Summary**

**Nested classes/interfaces inherited from class org.jdesktop.el.Expression**

`Expression.ResolvedProperty, Expression.Result`


**Constructor Summary**

`ValueExpressionImpl()`

`ValueExpressionImpl(java.lang.String expr,
Node node,
FunctionMapper fnMapper,
VariableMapper varMapper,
java.lang.Class expectedType)`


| **Method Summary** | |
| --- | --- |
| `boolean` | `equals(java.lang.Object obj)`             Determines whether the specified object is equal to this `Expression`. |
| `java.lang.Class` | `getExpectedType()`             Returns the type the result of the expression will be coerced to after evaluation. |
| `java.lang.String` | `getExpressionString()`             Returns the type the result of the expression will be coerced to after evaluation. |
| `Expression.Result` | `getResult(ELContext context, boolean trackResolvedObjects)` |
| `java.lang.Class` | `getType(ELContext context)`             Evaluates the expression relative to the provided context, and returns the most general type that is acceptable for an object to be passed as the `value` parameter in a future call to the `ValueExpression.setValue(org.jdesktop.el.ELContext, java.lang.Object)` method. |
| `java.lang.Object` | `getValue(ELContext context)`             Evaluates the expression relative to the provided context, and returns the resulting value. |
| `int` | `hashCode()`             Returns the hash code for this `Expression`. |
| `boolean` | `isLiteralText()`             Returns whether this expression was created from only literal text. |
| `boolean` | `isReadOnly(ELContext context)`             Evaluates the expression relative to the provided context, and returns `true` if a call to `ValueExpression.setValue(org.jdesktop.el.ELContext, java.lang.Object)` will always fail. |
| `void` | `readExternal(java.io.ObjectInput in)` |
| `void` | `setValue(ELContext context, java.lang.Object value)`             Evaluates the expression relative to the provided context, and sets the result to the provided value. |
| `java.lang.String` | `toString()` |
| `void` | `writeExternal(java.io.ObjectOutput out)` |

**Methods inherited from class org.jdesktop.el.ValueExpression**

`getSource, setSource`

**Methods inherited from class java.lang.Object**

`clone, finalize, getClass, notify, notifyAll, wait, wait, wait`

**Constructor Detail**

### ValueExpressionImpl

```
public ValueExpressionImpl()
```

---


### ValueExpressionImpl

```
public ValueExpressionImpl(java.lang.String expr,
                           Node node,
                           FunctionMapper fnMapper,
                           VariableMapper varMapper,
                           java.lang.Class expectedType)
```


**Method Detail**

### equals

```
public boolean equals(java.lang.Object obj)
```

:   **Description copied from class: `Expression`**
:   Determines whether the specified object is equal to this
    `Expression`.

    The result is `true` if and only if the argument is
    not `null`, is an `Expression` object that
    is the of the same type (`ValueExpression` or
    `MethodExpression`), and has an identical parsed
    representation.

    Note that two expressions can be equal if their expression
    Strings are different. For example, `${fn1:foo()}`
    and `${fn2:foo()}` are equal if their corresponding
    `FunctionMapper`s mapped `fn1:foo` and
    `fn2:foo` to the same method.

    :   **Specified by:**: `equals` in class `Expression`
    :   **Parameters:**: `obj` - the `Object` to test for equality. **Returns:**: `true` if `obj` equals this `Expression`; `false` otherwise. **See Also:**: `Hashtable`, `Object.equals(java.lang.Object)`

---


### getExpectedType

```
public java.lang.Class getExpectedType()
```

:   **Description copied from class: `ValueExpression`**
:   Returns the type the result of the expression will be coerced to
    after evaluation.

    :   **Specified by:**: `getExpectedType` in class `ValueExpression`
    :   **Returns:**: the `expectedType` passed to the `ExpressionFactory.createValueExpression` method that created this `ValueExpression`.

---


### getExpressionString

```
public java.lang.String getExpressionString()
```

:   Returns the type the result of the expression will be coerced to after
    evaluation.

    :   **Specified by:**: `getExpressionString` in class `Expression`
    :   **Returns:**: the `expectedType` passed to the `ExpressionFactory.createValueExpression` method that created this `ValueExpression`. **See Also:**: `javax.el.Expression#getExpressionString()`

---


### getType

```
public java.lang.Class getType(ELContext context)
                        throws PropertyNotFoundException,
                               ELException
```

:   **Description copied from class: `ValueExpression`**
:   Evaluates the expression relative to the provided context, and
    returns the most general type that is acceptable for an object to be
    passed as the `value` parameter in a future call
    to the `ValueExpression.setValue(org.jdesktop.el.ELContext, java.lang.Object)` method.

    This is not always the same as `getValue().getClass()`.
    For example, in the case of an expression that references an
    array element, the `getType` method will return the
    element type of the array, which might be a superclass of the type
    of the actual element that is currently in the specified
    array element.

    :   **Specified by:**: `getType` in class `ValueExpression`
    :   **Parameters:**: `context` - The context of this evaluation. **Returns:**: the most general acceptable type; otherwise undefined. **Throws:**: `PropertyNotFoundException` - if one of the property resolutions failed because a specified variable or property does not exist or is not readable.: `ELException` - if an exception was thrown while performing property or variable resolution. The thrown exception must be included as the cause property of this exception, if available.

---


### getValue

```
public java.lang.Object getValue(ELContext context)
                          throws PropertyNotFoundException,
                                 ELException
```

:   **Description copied from class: `ValueExpression`**
:   Evaluates the expression relative to the provided context, and
    returns the resulting value.

    The resulting value is automatically coerced to the type
    returned by `getExpectedType()`, which was
    provided to the `ExpressionFactory` when this
    expression was created.

    :   **Specified by:**: `getValue` in class `ValueExpression`
    :   **Parameters:**: `context` - The context of this evaluation. **Returns:**: The result of the expression evaluation. **Throws:**: `PropertyNotFoundException` - if one of the property resolutions failed because a specified variable or property does not exist or is not readable.: `ELException` - if an exception was thrown while performing property or variable resolution. The thrown exception must be included as the cause property of this exception, if available.

---


### getResult

```
public Expression.Result getResult(ELContext context,
                                   boolean trackResolvedObjects)
                            throws PropertyNotFoundException,
                                   ELException
```

:   **Overrides:**: `getResult` in class `ValueExpression`
:   **Throws:**: `PropertyNotFoundException`: `ELException`

---


### hashCode

```
public int hashCode()
```

:   **Description copied from class: `Expression`**
:   Returns the hash code for this `Expression`.

    See the note in the `Expression.equals(java.lang.Object)` method on how two expressions
    can be equal if their expression Strings are different. Recall that
    if two objects are equal according to the `equals(Object)`
    method, then calling the `hashCode` method on each of the
    two objects must produce the same integer result. Implementations must
    take special note and implement `hashCode` correctly.

    :   **Specified by:**: `hashCode` in class `Expression`
    :   **Returns:**: The hash code for this `Expression`. **See Also:**: `Expression.equals(java.lang.Object)`, `Hashtable`, `Object.hashCode()`

---


### isLiteralText

```
public boolean isLiteralText()
```

:   **Description copied from class: `Expression`**
:   Returns whether this expression was created from only literal text.

    This method must return `true` if and only if the
    expression string this expression was created from contained no
    unescaped EL delimeters (`${...}` or
    `#{...}`).

    :   **Specified by:**: `isLiteralText` in class `Expression`
    :   **Returns:**: `true` if this expression was created from only literal text; `false` otherwise.

---


### isReadOnly

```
public boolean isReadOnly(ELContext context)
                   throws PropertyNotFoundException,
                          ELException
```

:   **Description copied from class: `ValueExpression`**
:   Evaluates the expression relative to the provided context, and
    returns `true` if a call to `ValueExpression.setValue(org.jdesktop.el.ELContext, java.lang.Object)` will
    always fail.

    :   **Specified by:**: `isReadOnly` in class `ValueExpression`
    :   **Parameters:**: `context` - The context of this evaluation. **Returns:**: `true` if the expression is read-only or `false` if not. **Throws:**: `PropertyNotFoundException` - if one of the property resolutions failed because a specified variable or property does not exist or is not readable.: `ELException` - if an exception was thrown while performing property or variable resolution. The thrown exception must be included as the cause property of this exception, if available. \* @throws NullPointerException if context is `null`

---


### readExternal

```
public void readExternal(java.io.ObjectInput in)
                  throws java.io.IOException,
                         java.lang.ClassNotFoundException
```

:   **Specified by:**: `readExternal` in interface `java.io.Externalizable`
:   **Throws:**: `java.io.IOException`: `java.lang.ClassNotFoundException`

---


### setValue

```
public void setValue(ELContext context,
                     java.lang.Object value)
              throws PropertyNotFoundException,
                     PropertyNotWritableException,
                     ELException
```

:   **Description copied from class: `ValueExpression`**
:   Evaluates the expression relative to the provided context, and
    sets the result to the provided value.

    :   **Specified by:**: `setValue` in class `ValueExpression`
    :   **Parameters:**: `context` - The context of this evaluation.: `value` - The new value to be set. **Throws:**: `PropertyNotFoundException` - if one of the property resolutions failed because a specified variable or property does not exist or is not readable.: `PropertyNotWritableException` - if the final variable or property resolution failed because the specified variable or property is not writable.: `ELException` - if an exception was thrown while attempting to set the property or variable. The thrown exception must be included as the cause property of this exception, if available.

---


### writeExternal

```
public void writeExternal(java.io.ObjectOutput out)
                   throws java.io.IOException
```

:   **Specified by:**: `writeExternal` in interface `java.io.Externalizable`
:   **Throws:**: `java.io.IOException`

---


### toString

```
public java.lang.String toString()
```

:   **Overrides:**: `toString` in class `java.lang.Object`


---


|  |  |  |
| --- | --- | --- |
| **Overview** \| **Package** \| **Class** \| **Use** \| **Tree** \| **Deprecated** \| **Index** \| **Help** | |  |
| **PREV CLASS**   **NEXT CLASS** | **FRAMES**    **NO FRAMES**     **All Classes** |  |
| SUMMARY: NESTED \| FIELD \| CONSTR \| METHOD | DETAIL: FIELD \| CONSTR \| METHOD |  |


---
